# From Vascular Smooth Muscle Cells to Folliculogenesis: What About Vasorin?

**DOI:** 10.3389/fmed.2018.00335

**Published:** 2018-12-04

**Authors:** Anne-Laure Bonnet, Catherine Chaussain, Isabelle Broutin, Gaël Y. Rochefort, Heinrich Schrewe, Céline Gaucher

**Affiliations:** ^1^EA 2496 Orofacial Pathologies, Imaging and Biotherapies, Dental School Faculty, University Paris Descartes and Life Imaging Platform (PIV), Montrouge, France; ^2^Department of Odontology, University Hospitals Charles Foix, PNVS, and Henri Mondor, AP-HP, Paris, France; ^3^Laboratoire de Cristallographie et RMN Biologiques (UMR 8015, CNRS), Pharmacy Faculty, University Paris Descartes, USPC, Paris, France; ^4^Department of Developmental Genetics, Max Planck Institute for Molecular Genetics, Berlin, Germany

**Keywords:** Slitl2, TGF-β, Notch1, biomarker, development, pathophysiology

## Abstract

First described in 1988, vasorin (VASN) is a transmembrane glycoprotein expressed during early mouse development, and with a less extent, in various organs and tissues (e.g., kidney, aorta, and brain) postnatally. *Vasn* KO mice die after 3 weeks of life from unknown cause(s). No human disease has been associated with variants of this gene so far, but VASN seems to be a potential biomarker for nephropathies and tumorigenesis. Its interactions with the TGF-β and Notch1 pathways offer the most serious assumptions regarding VASN functions. In this review, we will describe current knowledge about this glycoprotein and discuss its implication in various organ pathophysiology.

## Introduction

Vasorin (VASN), a cell surface glycoprotein of 673 amino acids (aa), is encoded by the *VASN* gene also named Slit-like 2 (*Slitl2*) due to its strong homologies with the slit family of important signaling molecules. Vasorin was identified by various screens in several vertebrates including rodents (*Mus musculus* and *Rattus norvegicus*) (UniProt Q9CZT5 and D3ZAE6), zebrafish (*Danio rerio*) (UniProt A4QNV9), and humans (*Homo sapiens*) (UniProt Q6EMK4). Located on chromosome 16 in human and mouse, it is encoded by two exons separated by a large intronic sequence (Supplementary Figure 1A). Vasorin is highly conserved at the DNA and protein levels; alignments of the coding region reveal an overall identity of more than 95 and 83% at the amino acid level between rodent and human homologs, respectively (Supplementary Figure 1B) (1). This high degree of similarity suggests a highly conserved function of the protein throughout evolution, but until now, no human disease or phenotype has been directly associated to variants of the *VASN* gene. In 2004, Ikeda et al. published the first study about VASN reporting its localization in adult human tissues and suggesting a role in the TGF-β pathway (2). Fourteen years later, the roles of VASN/Vasn in development or in the pathophysiology of adult tissues or organs remains unelucidated although some clues are emerging. This review aims at reporting current knowledge on Vasn/VASN and more specifically at describing the main pathways that have been associated to this transmembrane protein.

## Vasorin Structure and Feature Prediction

**Figure 1:**
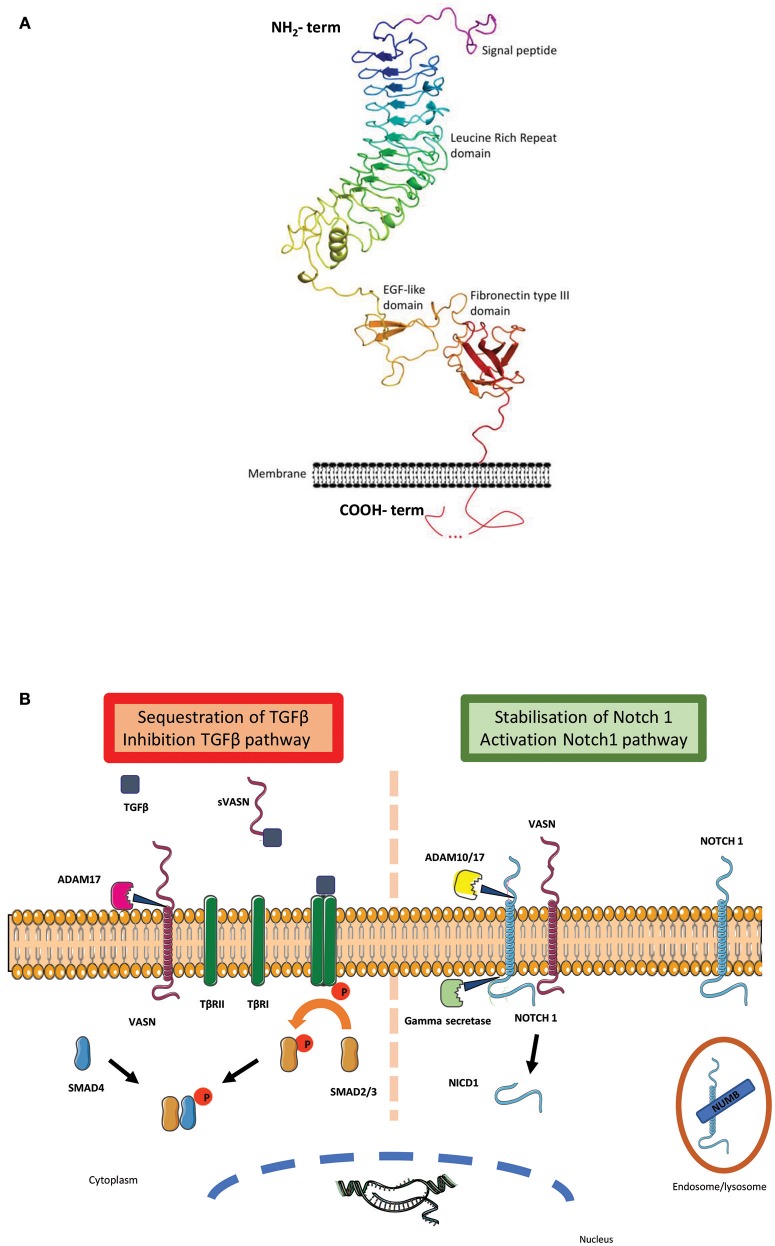
**(A)** Vasorin 3D structure: Cartoon representation of the structure model of Vasn predicted by RaptorX (http://raptorx.uchicago.edu/). It is represented in rainbow color from the N-terminus to the C-terminus, at the exception of the predicted signal peptide (http://www.cbs.dtu.dk/services/SignalP/) colored in purple. The transmembrane fragment predicted by TMHMM (http://www.cbs.dtu.dk/services/TMHMM/) is localized at its place on the schematic membrane. The different predicted domains are indicated along the structure. The intracellular carboxy-terminal peptide with unknown structure is represented with a dotted line (4). **(B)** Vasorin major pathways: Vasn was first described as an inhibitor of TGF-β pathway: the metalloprotease ADAM 17 cleaves VASN and releases the soluble extracellular part of vasorin (sVASN). TGF-β binds to sVASN instead of TGFβRII. The hetero-tetrameric complex between TGF-β1 and−2 is not formed and the sequence of intra-cellular phosphorylation does not occur [adapted from (5)]. A second pathway was recently discovered showing that Notch1 can be stabilized at the membrane by VASN, thus escaping Numb (an inhibitor of the activation of Notch signaling) mediated endocytosis and lysosomal degradation. This bonding allows both extracellular cleavage by ADAM 10/17 and intracellular cleavage by the gamma secretase to cleave Notch1 intracellular peptide NICD1, which translocates to the nucleus to form a transcription complex [adapted from (6)].

Vasorin (VASN/Vasn) is a typical, single-pass, type I transmembrane protein of ~110 kDa. Based on its sequence analysis by Blast and by the RaptorX modeling program (3), a prediction of its tri-dimensional structure has been performed, using mainly neuronal adhesion molecules (Netrin-G, LRRTM2, and NCAM2) as probes, leading to the following description. Its extracellular amino-terminal domain contains a putative hydrophobic signal peptide, one leucine-rich repeat (LRR) region, comprising 11 LRR repeats, flanked by an amino- and a carboxy-terminal LRR-flank motif, one epidermal growth factor (EGF)-like repeat, and one fibronectin type III (FNIII) domain (Figure 1A; Supplementary Figure 1). These regions are followed by a highly hydrophobic stretch of amino acids that are predicted to be a single pass transmembrane segment. The intracellular carboxy-terminal peptide, of ~80 amino-acid residues, shows no similarity to any known structure so far. The combination of LRR regions (4–7 repeats depending on the family's members) and EGF domain is conserved within the Slit family of proteins (7). Interestingly, this serial repetition of the LRR motifs is known to form non-globular, horseshoe structure, allowing tight protein-protein interaction. The concave sides of those structures are specifically involved in the binding with the Ig domains of the Robos receptors (8, 9). It is important to note that, with its 11 LRR repetition motifs, Vasn offers a large extracellular binding site.

Discussing Vasn structure also raises the possibility of the existence of a cleavage site close to the EGF region. In Slit proteins, this cleavage occurs within the EGF repeats, after the arginine (R) of a VLPR motif, releasing the N-terminal active isoform (8). Malapeira et al. could show that in breast cancer cell lines, the N-terminal part of Vasn was cleaved by ADAM-17 and that only this cleaved form of Vasn was active for TGF-β trapping (5). This observation was confirmed in the human hepatocellular carcinoma (HCC) cell line (Hep G2) by Li et al. (10). Indeed, many arginine residues are present within or around the EGF motif, especially a VTPR motif, the most similar motif to the VLPR, located close to the FNIII domain in the Vasn sequence.

## Vasorin Expression and Localization

Vasn/VASN expression pattern suggests major role(s) from embryonic development to adulthood (Table 1). Whole mount *in situ* hybridization in zebrafish, embryos revealed that Vasorin mRNA is detected as early as the gastrula stage and rapidly spreads along the dorsal-ventral axis, and later along the antero-posterior axis with a prominent expression observed in the brain primordium during the protruding mouth stage, and in the central neural system (1). In rodents, a similar pattern is observed between E8.5 and E11.5 in murine embryos, with strong expression in the hindbrain and the midline of the neural tube. In addition, a strong expression is observed in the first branchial arch and the forelimb and hindlimb buds (20). In this latest study, a reporter gene expression was used in Vasn^LacZ^ and in Vasn^Venus^ transgenic mice and confirmed the endogenous Vasn expression. From E10.5 onwards, the reporter signal is mainly found around the developing heart, lungs, kidney, testis/ovary, and skeletal system and increases at subsequent stages. At E17.5, an intense reporter signal is precisely observed in the ossification region of long bones, in vascular smooth muscle cell-rich arterial vessels, in the mucosa lining the gastric fundus and in the spleen capsule, along with previously described organs/tissues.

**Table 1:** Vasorin expression and methods of localization.

**Tissues**	**Methods**	**References**
**HUMAN**		
Aorta, Kidney, Placenta	NB	(2)
Urine of TBMN patients	LC-MS/MS; WB	(11)
Urine of diabetic patients	LC-MS/MS	(12)
Different cancer cell lines	LC-MS/MS; WB	(5)
12 different tumor cell lines	LC-MS/MS; MALDI TOF	(13)
Synovial fluid	LC-MS/MS	(14)
HepG2 cells	ICC; IF; WB	(15)
HepG2 cells Serum HCC and Hepatitis patients	MALDI TOF; EMSA SELEX; ELISA	(16)
Follicular fluid from fertile women	LC-MS/MS	(17)
Teeth	LC-MS/MS; WB	(18)
Brain, GSCs	ICC; IF; WB; FC; ChIP; RNA Seq; IHC	(6)
HepG2 cells	ELISA; WB	(10)
**MOUSE**		
Embryo, kidney, aorta, ovaries	ISH; IHC; NB; WB	(2)
MEF; all tissues	WB; NB; qPCR; IHC	(19)
Embryo	X gal staining	(20)
Ovaries	RT qPCR; WB	(21)
**RAT**		
VSMCs	IHC; NB; WB	(2)
**HAMSTER**		
CHO cells	IHC; NB; WB	(2)
**ZEBRAFISH**		
Embryo	WisH	(1)
Larvae	ISH; IHC	(22)

*ChIP, Chromatin Immuno-Precipitation; ELISA, Enzyme Linked ImmunoSorbent Assay; EMSA SELEX, Electrophoretic Mobility Shift Assay-Systematic Evolution of Ligands by Exponential Enrichment; FC, Flow Cytometry; IF, ImmunoFluorescence; IHC, Immuno Histo Chemistry; ICC, Immuno Cytochemistry; LC-MS/MS, Liquid Chromatography coupled to tandem Mass Spectrometry; NB, Northern Blot; ISH, In Situ Hybridization; MALDI TOF, Matrix Assisted Laser Desorption Ionization-Time Of Flight; RT qPCR, Reverse Transcription Quantitative Polymerase Chain Reaction; WisH, Whole mount in situ Hybridization, WB, Western Blot; CHO, Chinese Hamster Ovary; GSCs, Glioblastoma Stem cells; HCC, Hepatocellular Carcinoma; HepG2, Human hepatocellular carcinoma cells; MEF, Mouse Embryonic Fibroblasts; TMBN, Thin Basement Membrane Nephropathy; VSMCs, Vascular Smooth Muscle Cell*.

These approaches revealed a widespread expression of Vasn from early developmental stage narrowing as development progresses. *In situ* hybridization on adult mice confirmed Vasn expression in the coronary artery, aorta, kidney (2), and in ovarian follicles (21). Northern blot analysis on adult mice tissues further confirmed Vasn expression in heart, kidney, lung, liver, and testis (19).

In human, VASN was identified by Northern blot in aorta, with a high expression, compared to moderate levels in kidney, liver, and placenta (2). It was also detected in vascular smooth muscle cells (VSMCs) (2), human umbilical vein endothelial cells (HUVECs) (15), and in permanent periodontal ligament cells (18). Finally, less relevant but still notable, a proteomic approach on healthy fertile women ovum donors identified VASN in the human follicular fluid (17).

At the cellular level, Ikeda et al. have first reported Vasn expression at the cell-surface membrane, using Chines Hamster Ovary (CHO) cells expressing a human VASN-flag-tagged protein (2). This localization was confirmed in breast cancer cell line MCF7 (5), in Hep G2 (10, 16), and in human glioma stem like cells (GSCs) (6). In parallel, the presence of a secreted extracellular peptide has been highlighted by Malapeira et al. (5), Li et al. (10), Huang et al. (15), and Man et al. (6) but the precise function of this secreted peptides remains to be elucidated. Only one study described an intracellular form of vasorin (called ATIA for Anti TNFα Induced Apoptosis in this study) translocating to the mitochondria in mouse embryonic fibroblasts (19).

One notable point confirmed by most of studies is the molecular weight of VASN/Vasn around 110 kDa revealed by western blot (2, 5, 10). Some authors propose that the N- or O- glycosylation of the protein could explain such a difference with the expected molecular weight at 72 kDa (2, 19). Only one study presented a different result with a total VASN expression band at around 80 kDa after western blotting (11); the extracellular soluble form has been reported to be around 90 kDa (5).

## Vasorin Partners and Pathways

In the primary study, Ikeda et al. have investigated VSMCs and a rat carotid arterial balloon-injury model to demonstrate that Vasn directly bound to transforming growth factor TGF-β1, TGF-β2, and TGF-β3 at the membrane surface or at the extracellular level and inhibited TGF-β signaling *in vitro* (2). *In vivo*, Vasn expression was down-regulated after vascular injury whereas the expression of several cytokines, including TGF-β, was up-regulated and the ratio of TGF-β to Vasn was increased. In addition, Vasn administration after injury dramatically reduced the neointimal formation, at least in part by modulating TGF-β signaling in the vessel wall as shown by the decrease of Smad2 phosphorylation. Taken together, these data suggested that the down-regulation of Vasn induced by acute vascular injury contributed to the fibroproliferative response to vascular injury.

Following these findings, Malapeira et al. reported that only the soluble extracellular part of vasorin (sVASN) functioned as a trap for TGFβ and confirmed a direct inhibiting effect of sVASN on TGFβ signaling pathway, manifesting by a decrease in pSmad levels in response to TGFβ treatment (5). In addition, they demonstrated that the metalloprotease ADAM17 was able to cleave VASN—although not the only one able to do so—and that in their CHO cells model it was the major protease involved in this shedding (Figure 1B). These results were reproduced in the breast cancer cell lines MCF7 and A459 (6) and in addition it was shown that inhibition of ADAM17 by a metalloprotease inhibitor—BB94—led to the upregulation of TGF-β signaling.

In 2011, Choksi et al. explored vasorin in a hypoxia and TNFα-induced apoptosis context (19) and demonstrated that ATIA (VASN) was highly expressed in human glioblastoma and its inhibition allowed hypoxia-mediated apoptosis of cells. They hypothesized that ATIA would be a hypoxia inducible factor (HIF-1) target and generated a partial ATIA KO model targeting the supposed mitochondrial addressing peptide of intracellular VASN. In their mouse and MEF models, ATIA appeared to protect cells against TNFα-induced apoptosis, at least partially through mitochondrial-ATIA and thioredoxin (TRX2) interactions. Looking further on this HIF1 pathway and using GSCs, Man et al. confirmed that Vasorin expression was up-regulated by hypoxia, as shown by its co-activation with many other hypoxia response genes, and that Vasorin was mandatory to maintain GSCs under hypoxic conditions (6). This study demonstrated that Notch1 was one major partner in this function and that Vasorin competed Numb (the Notch pathway inhibitor) for Notch1 binding, thus preventing its lysosomal degradation. Hence, when vasorin is present, it appears to decrease Notch1 turnover and increase Notch1 nuclear signaling, thus allowing GSCs renewal and tumorigenic properties.

### Vasorin as a Biomarker

Nowadays, diagnosing early stages of pathologies is a major challenge. Numerous studies are searching for non-invasive methods to discriminate healthy vs. ill individuals or to follow, on an individual based strategy, disease/treatments evolution (23).

Along this line, vasorin has been identified as a potential biomarker of severe progressive nephropathies such as Immunoglobulin A nephropathy (IgAN), thin basement membrane nephropathy (TBMN), or diabetic nephropathy (DN). Vasorin was detected in higher amount by nano LC-MS/MS in the urinary exosome of patients with TBMN compared to that of patients with IgAN as well as control patients, and confirmed by western blot analysis (11). Using nano LC-MS/MS but on total urine sample proteome extracts, Samavat et al. also reported the presence of VASN in the urine of patients with IgAN (24). VASN has also been isolated as a glycoprotein in plasma of patients with diabetic nephropathies. Interestingly, VASN was upregulated in diabetic patients with nephropathy in contrast to diabetic patients without nephropathy (12, 24). In addition, a study also described VASN in synovial fluid of patients with osteoarthritis (14).

Following this idea but in another domain, Vasorin was also identified in a few studies looking for cancer biomarkers. In 2011, Caccia et al. reported the analysis of 12 different tumor cell lines, aiming to identify by proteomic assay specific proteins under- or over-expressed in cell secretomes (13). Using the thyroid cancer cell line TPC-1, they identified VASN as one of the 3 specifically under-expressed proteins after inhibiting proliferation treatment in the cell cultures.

In 2011 also, Choksi et al. proposed that ATIA could be a biomarker for brain cancer due its significant overexpression in brain tumor cell-lines (glioblastoma and astrocytoma) (19). In 2018, the same team showed that VASN expression was increasing along with gliomas aggressiveness (6).

In 2015, Li et al. reported that VASN was a prospective biomarker of HCC and a potential therapeutic target for this cancer (16). Using subtractive-EMSA-SELEX and MALDI-TOF MS assay they verified that VASN was highly expressed in alpha-fetoprotein (AFP, a classical serological biomarker for liver cancer) negative sera of 100 cases of HCC patients with extrahepatic metastases compared with 97 cases of healthy controls, and 129 cases of hepatitis patients. In addition, using a siRNA based VASN knockdown approach in the cancerous Hep-G2 cell line, they showed a decreased cell proliferation, an increased apoptosis and a reduced migration compared within normal L02 cells. Finally, they identified 2 miRNA on the 7 targeting vasorin sequence (miR145 and miR146a), which were expressed in Hep G2, SMMC7721, and L02 cell lines but were negatively correlated to VASN mRNA level, and whose transfection in Hep G2 cells down-regulated VASN expression, and promoted apoptosis with decreased cell proliferation and migration.

### A Little Bit Further Into Vasorin Role(s) in Pathophysiology

Since the first publication in 2004, the major roles of vasorin appear to be related to cell migration and proliferation/differentiation. This fits well with its structural homologies with the Slit family and its expression in specialized cells and in remodeling organs/tissues.

Considering VASN strong expression in the vascular system and its role in tumorigenesis, Huang et al. hypothesized that vasorin might play a pivotal role in tumorigenesis and vasculogenesis (15). They explored vasorin transfer from Hep G2 to (HUVECs) via an exosome/endocytosis process and demonstrated not only the existence of this transfer, but also that VASN enhanced HUVEC migration. This indicates that VASN could act as a mediator for cancer cells toward their environment, promoting endothelial cells invasion and consecutively tumor development and metastasis.

Along the idea of TGF-β pathway central role, Rimon-Dahari et al. explored the role of Vasn in folliculogenesis (21). They first reported vasorin expression in ovaries at all the follicular stages and used Vasn systemic KO mice to analyse mice fertility and folliculogenesis. They noticed an enhanced ovulation under hormonal stimulation in KO mice as well as in transplanted KO ovaries in wild type females. However, there was no difference regarding fertility. Vasn-deficient females displayed a significantly lower number of primordial follicles as well as atretic antral follicles compared to wild type mice. The absence of vasorin was also correlated with an overactivated TGF-β pathway in KO mice, evidenced by an increase in pSmad2 levels in KO ovaries after hormonal stimulation. Although exploring a new aspect in vasorin functions, this study did not show the existence of a direct link between the absence of Vasn and TGF-β modulation.

## Conclusion

Considering data on the expression of Vasorin in humans or in animal models, one may not be surprised to encounter an altered expression of this protein in human diseases such as nephropathies or cancers. In this context, the use of sVASN as a biomarker seems promising but requires further investigations. Nevertheless, data reporting the physiological distribution and elimination of this secreted form are still missing. Li et al. as continuity of their research on liver cancer (16), identified two mimotopes of sVASN that might be useful to address these questions, as well as to develop biological therapeutic targets (10). Vasorin links with the TGF-β pathway has also led to an analysis of its involvement in tumorigenesis, with strong evidence for a role in cell migration and proliferation. It seems that the main roles of vasorin are driven by its extracellular domain under the control of extracellular proteases such as ADAM 10 and 17 (5, 6). More recently, an exciting interaction with the Notch1 pathway has been unraveled in the context of cancer (6). Considering this slow and step-by-step progression, one may certainly say that there is still a great deal to discover about vasorin. The study of mutant mouse lines will enable either a global, or an organ-restricted, approach to better understand the impact of Vasorin deletion or activation. Studying total knock-out mice for Vasn may confirm a deleterious vascular and/or skeletal phenotype, which could be expected considering the strong Vasn embryonic expression. These models could also reveal other affected organs/tissues in childhood or adulthood. In addition, the kidney appears as a promising target for specific conditional mouse line, but the exact localization of Vasorin in nephrotic specialized cells (tubules cells, podocytes) remains to be defined. All this knowledge opens and will open many other avenues for further research.

## Author Contributions

CG, A-LB, GR, and CC drafted the manuscript. A-LB wrote the first draft of the manuscript and figures. IB, GR, and CG wrote sections and figures of the manuscript. CC and HS critically revised the manuscript. All authors contributed to manuscript revision, read, and approved the submitted version.

### Conflict of Interest Statement

The authors declare that the research was conducted in the absence of any commercial or financial relationships that could be construed as a potential conflict of interest.
