# Supplementary material for: From Vascular Smooth Muscle Cells to Folliculogenesis: What About Vasorin?
Source: Front Med (Lausanne). 2018 Dec 4;5:335. doi: 10.3389/fmed.2018.00335 (PMC6288187; doi:10.3389/fmed.2018.00335)
Supplement: Supplementary file 1 [file Data_Sheet_1.docx]

**A)**

**
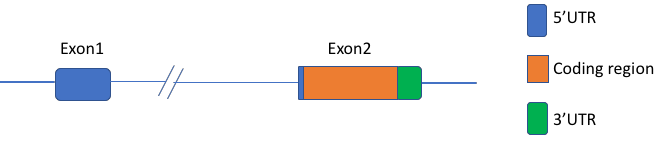
**

**B)**

VASN_HUMAN      MCSRVP-LLLPLLLLLALGPGVQGCPSGCQCSQPQTVFCTARQGTTVPRDVPPDTVGLYV 59

VASN_MOUSE      MHSRSCLPPLLLLLLVLLGSGVQGCPSGCQCNQPQTVFCTARQGTTVPRDVPPDTVGLYI 60

                * **     * ****: ** ***********.***************************:

VASN_HUMAN      FENGITMLDAGSFAGLPGLQLLDLSQNQIASLPSGVFQPLANLSNLDLTANRLHEITNET 119

VASN_MOUSE      FENGITTLDVGCFAGLPGLQLLDLSQNQITSLPGGIFQPLVNLSNLDLTANKLHEISNET 120

                ****** **.*.*****************:***.*:****.**********:****:***

VASN_HUMAN      FRGLRRLERLYLGKNRIRHIQPGAFDTLDRLLELKLQDNELRALPPLRLPRLLLLDLSHN 179

VASN_MOUSE      FRGLRRLERLYLGKNRIRHIQPGAFDALDRLLELKLPDNELRVLPPLHLPRLLLLDLSHN 180

                **************************:********* *****.****:************

VASN_HUMAN      SLLALEPGILDTANVEALRLAGLGLQQLDEGLFSRLRNLHDLDVSDNQLERVPPVIRGLR 239

VASN_MOUSE      SIPALEAGILDTANVEALRLAGLGLRQLDEGLFGRLLNLHDLDVSDNQLEHMPSVIQGLR 240

                *: *** ******************:*******.** *************::* **:***

VASN_HUMAN      GLTRLRLAGNTRIAQLRPEDLAGLAALQELDVSNLSLQALPGDLSGLFPRLRLLAAARNP 299

VASN_MOUSE      GLTRLRLAGNTRIAQIRPEDLAGLTALQELDVSNLSLQALPSDLSSLFPRLRLLAAARNP 300

                ***************:********:****************.***.**************

VASN_HUMAN      FNCVCPLSWFGPWVRESHVTLASPEETRCHFPPKNAGRLLLELDYADFGCPATTTTATVP 359

VASN_MOUSE      FNCLCPLSWFGPWVRENHVVLASPEETRCHFPPKNAGRLLLDLDYADFGCPVTTTTATVP 360

                ***:************.**.*********************:*********.********

VASN_HUMAN      TTRPVVREPTALSSSLAPTWLSPTEPATEAPSPPSTAPPTVGPVPQPQDCPPSTCLNGGT 419

VASN_MOUSE      TIRSTIREPTLSTSSQAPTWPSLTEPTTQASTVLSTAPPTMRPAPQPQDCPASICLNGGS 420

                * * .:****  :** **** * ***:*:* :  ******: *.******* * *****:

VASN_HUMAN      CHLGTRHHLACLCPEGFTGLYCESQMGQGTRPSPTPVTPRPPRSLTLGIEPVSPTSLRVG 479

VASN_MOUSE      CRLGARHHWECLCPEGFIGLYCESPVEQGMKPSSIPDTPRPPPLLPLSIEPVSPTSLRVK 480

                *:**:***  ******* ****** : ** :**  * *****  * *.***********

VASN_HUMAN      LQRYLQGSSVQLRSLRLTYRNLSGPDKRLVTLRLPASLAEYTVTQLRPNATYSVCVMPLG 539

VASN_MOUSE      LQRYLQGNTVQLRSLRLTYRNLSGPDKRLVTLRLPASLAEYTVTQLRPNATYSICVTPLG 540

                *******.:********************************************:** ***

VASN_HUMAN      PGRVPEGEEACGEAHTPPAVHSNHAPVTQAREGNLPLLIAPALAAVLLAALAAVGAAYCV 599

VASN_MOUSE      AGRTPEGEEACGEANTSQAVRSNHAPVTQAREGNLPLLIAPALAAVLLAVLAAAGAAYCV 600

                 **.**********:*  **:****************************.***.******

VASN_HUMAN      RRGRAMAAAAQDKGQVGPGAGPLELEGVKVPLEPGPKATEGGGEALPSGSECEVPLMGFP 659

VASN_MOUSE      RRARATS-TAQDKGQVGPGTGPLELEGVKAPLEPGSKATEGGGEALSGGPECEVPLMGYP 659

                **.** : :**********:*********.***** ********** .* ********:*

VASN_HUMAN      GPGLQSPLHAKPYI 673

VASN_MOUSE      GPSLQGVLPAKHYI 673

                **.**. * ** **

Supplementary Figure 1 **A)** Vasorin gene scheme: *Vasn* mouse NC_00082.6 and *VASN* human NC_00016.10; **B)** VASN/Vasn alignment. The different domains are highlighted with the same colours as in Figure 1: signal peptide, Leucine-Rich Repeat (LRR), EGF-like domain, Fibronectin type III (FN3) domain, Transmembrane segment (TM)

On the bottom line: * identity; : residues with conserved properties; . residues with few conserved properties
